# Annotated image dataset with different stages of European pear rust for UAV-based automated symptom detection in orchards

**DOI:** 10.1016/j.dib.2025.111271

**Published:** 2025-01-03

**Authors:** Virginia Maß, Pendar Alirezazadeh, Johannes Seidl-Schulz, Matthias Leipnitz, Eric Fritzsche, Rasheed Ali Adam Ibraheem, Martin Geyer, Michael Pflanz, Stefanie Reim

**Affiliations:** a Leibniz Institute for Agricultural Engineering and Bioeconomy^1^, Department Agromechatronics, Potsdam, Germany; b Julius Kühn-Institute, Federal Research Centre for Cultivated Plants^2^, Institute for Breeding Research on Fruit Crops, Dresden, Germany; c geo-konzept, Gesellschaft für Umweltplanungssyteme mbH, Adelschlag, Germany

**Keywords:** Yolo, *Gymnosporangium sabinae*, Phenotyping, Drone monitoring, Machine learning, Object detection

## Abstract

The evaluation of fruit genetic resources regarding a resistance to pathogens is an essential basis for subsequent selection in fruit breeding. Both genetic analysis and phenotyping of defined traits are important tools and provide decision data in the evaluation process. However, the phenotyping of plants is often carried out ‘by hand’ and remains the bottleneck in fruit breeding and fruit growing. The development of a digital and UAV (unmanned aerial vehicle)-based phenotyping method for the assessment of genotype-specific susceptibility or resistance against diseases in orchards would significantly increase the efficiency of plant breeding. In this framework, a workflow for drone-based monitoring of pathogens in orchards was developed using the European pear rust (*Gymnosporangium sabinae*) as model pathogen. Pear rust is widespread in orchards and causes conspicuous, clearly visible, yellow to orange-colored disease symptoms.

In this paper, we provide a dataset with expert-annotated high-resolution RGB images with pear rust symptoms. For data collection, ten UAV-flight campaigns were realized between 2021 and 2023 under various weather conditions and with different flight parameters in the experimental orchard of the Julius Kühn-Institute for Breeding Research on Fruit Crops in Dresden-Pillnitz (Germany). 1394 images were captured of different pear genotypes, including varieties, wild species and progeny from breeding. The dataset contains manually labelled images with a size of 768 × 768 pixels of leaves infected with pear rust at different stages of development, labelled as class GYMNSA, as well as background images without symptoms. Each leaf with pear rust symptoms was annotated with the drawing method by two points (bounding boxes) using the Computer Vision Annotation Tool (CVAT, v1.1.0) [1] and presented in YOLO 1.1 file format (.txt files). A total of 584 annotated images and 162 background images, organized into a training and validation set, are included in the GYMNSA dataset. This GYMNSA dataset can be used as a resource for researchers and developers working on drone-based plant disease monitoring systems.

Specifications Table

**Table:** 

Subject	Computer Science; Computer Vision and Pattern Recognition
Specific subject area	The dataset contains cropped and annotated UAV image files of pear rust infected leaves from the experimental orchard for computer vision and pattern recognition applications.
Data format	Raw. Filtered. Pre Processed.
Type of data	Cropped 2D RGB digital image files (.JPG). Cropped size: 3 × 768 × 768 pixels. YOLO 1.1 annotation files format (.txt) Table of the image metadata (.xlsx), using the ExifTool software (v12.3.8.0) [2] Table of the annotation metadata (.csv) Table of training annotations (.csv) Table of validation annotations (.csv)
Data collection	The raw UAV-RGB images were collected using the quadcopters DJI Phantom 4 Pro V2.0 (P4P, v01.08.1719) and DJI Matrice 300 RTK with Zenmuse P1 (v03.00.01.04, v07.00.01.10) camera. DJI GS Pro software (v2.0.16) and the DJI Pilot PE (v1.8.0) was used for the flight planning. The images were taken every 3 seconds during a flight speed of 1 meter per second. The flights were carried out with a front and side overlap of about 75 to 90 % at a flight altitude of approximately 5 to 12 meters above the ground. The average ground sampling distance (GSD) was 0.17 cm per pixel. The images were cropped into a size of 768 × 768 pixels without overlapping using the Python (v3.11.5) [3] library Pillow (v9.5.0) [4]. The annotation tool CVAT (v1.1.0) [1] was used for the 2-point bounding box annotation.
Data source location	The data source location was the experimental orchard of the Julius Kühn-Institute (JKI - Federal Research Centre for Cultivated Plants) at the Institute for Breeding Research on Fruit Crops located in Dresden-Pillnitz (Germany) [51°00ʹ01"N 13°53ʹ12"E].
Data accessibility	Repository name: Mendeley Data Data identification number: 10.17632/44kjgc4gkc.1 Direct URL to data: https://data.mendeley.com/datasets/44kjgc4gkc/1

## 1 Value of the Data

• These data were collected on an approximately 1.6 ha experimental field with over 1000 different pear genotypes (breeding material and genetic resources of pear varieties and species) and presents a wide spectrum of phenotypic characteristics of pear rust infections at different stages of development on morphologically different pear genotypes.
• A digital phenotyping method of pathogens using different genotypes will provide information on the characteristics and resistances and complement the manual phenotyping methods.
• Plant breeders and scientists can use the dataset to test and refine object detection using the model pathogen *Gymnosporangium sabinae* in the field of precision agriculture and the monitoring of plant diseases via UAVs.
• Early identification of the phenotypic characteristics of pathogens through UAV monitoring should help to facilitate the targeted use of plant protection products.

## 2 Background

The use of various plant protection products during the growing season is necessary to combat plant diseases, as they represent major economic risks in agricultural production [5]. As the use of plant protection products can be harmful to the environment and is restricted by legal regulations, the breeding of tolerant or resistant varieties is a necessity in agricultural research. In order to identify genotypes that are tolerant or resistant to the respective disease, the evaluation of genetic resources is a prerequisite. In addition to the development of genetic methods (e.g. molecular markers), the phenotyping of plants as a research tool contributes to improving the efficiency of breeding work [6,7]. Established methods of disease monitoring are based on manual assessments with punctual measurements. These are expensive, labour-intensive and thus be carried out only for selected plants and for a limited time. A solution would be the development of high-throughput digital phenotyping techniques to enable rapid and effective assessment of a wide range of genetic resources [8,9].

In order to utilise the capabilities of low-cost UAV technology for monitoring and mapping using high-resolution RGB images, the creation of the GYMNSA dataset with the specific symptoms of the model pathogen *Gymnosporangium sabinae* is mandatory.

## 3 Data Description

**Fig. 1:**
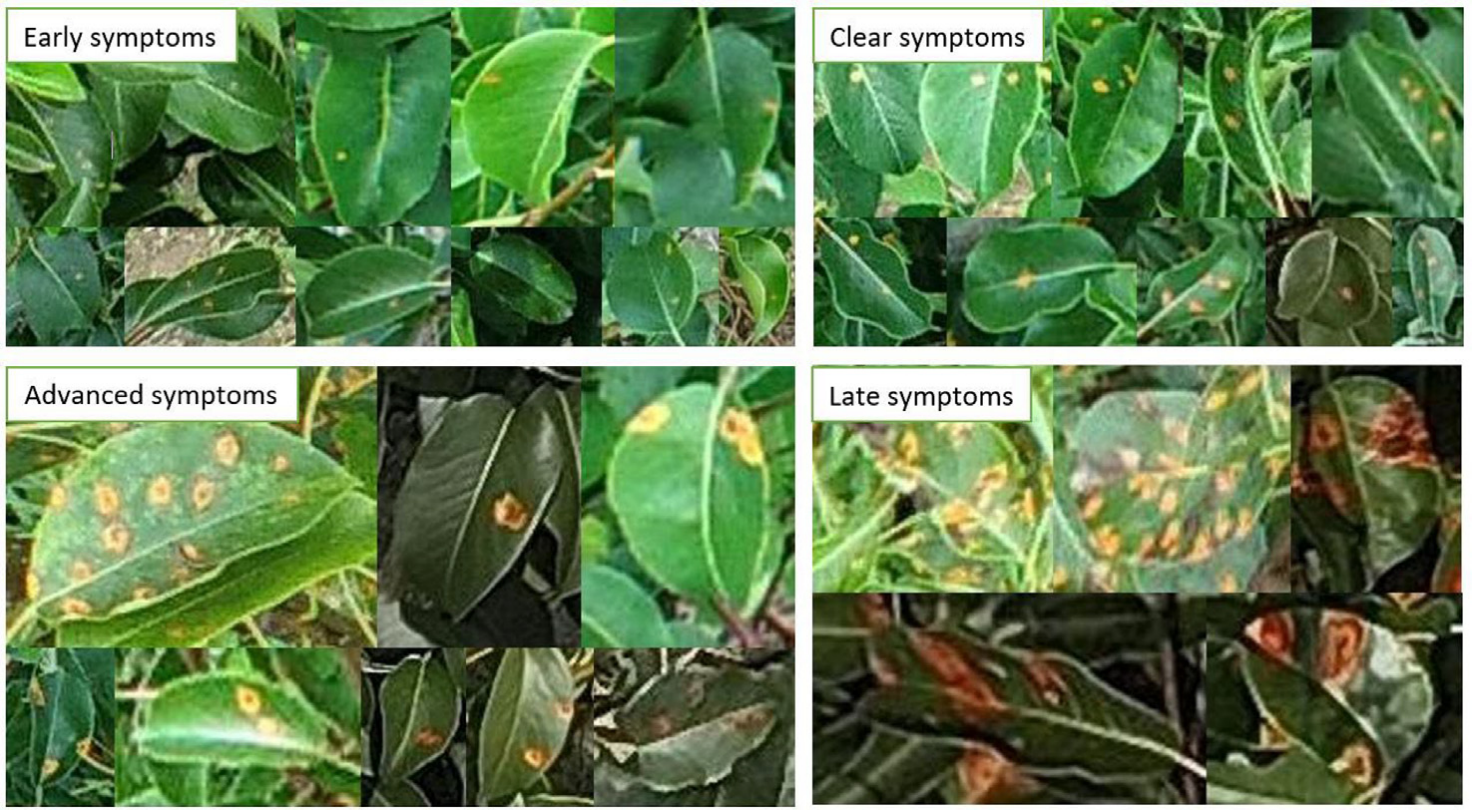
Selected sections of infection stages of European pear rust symptoms using images from the GYMNSA dataset. Early symptoms: Small yellow to light orange spots. Clear symptoms: Distinct light orange-orange spots that look wet-slimy. No spermogonia (black spots) visible. Advanced symptoms: Orange-coloured spots with spermogonia. Spots look dry. Leaf underside with brownish tissue growth from which aecidia develop. Late symptoms: Reddish large spots with spermogonia. Underside of leaf with brown tissue growth from which the aecidia develop [10].

The aim was to represent the diversity of disease symptoms in over 1000 pear genotypes. The experimental orchard of the Julius Kühn-Institute (JKI) at the Institute for Breeding Research on Fruit Crops in Dresden-Pillnitz (Germany) served as the data source. 231 RGB UAV images with a resolution of 5472  ×  3648 pixels (DJI P4P) and 8192  ×  5460 pixels (DJI Matrice 300 RTK - Zenmuse P1 camera) were used to create this dataset. Both high-resolution nadir and oblique images were included in the dataset. The flight campaigns were realised by the company geo-konzept and the JKI. The drone flights took place at ten different times within the vegetation period and at different infection stages (Fig. 1), different weather conditions and with different flight parameters.

The resulting 1394 original images were carefully checked for quality and the presence of disease symptoms in order to crop them in pre-processing to a pixel-based size of 768  ×  768 pixels without overlapping and save them in JPG format. After manual sorting, a total of 584 cropped images were selected for the annotation work. In addition, 162 cropped background images (768 × 768 pixels) without disease symptoms and with image details that could be mistaken for pear rust symptoms were added (Table 1).

**Table 1:** The number of selected original RGB images and cropped images (768  ×  768 pixels) used for annotation and background as well as the number of annotations according to date, camera system and relative altitude above ground (starting point). The original images have a resolution of 5472  ×  3648 pixel (DJI P4P) and 8192  ×  5460 pixel (* Zenmuse P1 camera).

Date	Drone / Camera-system	Relative Altitude [meter]	Original images		Cropped images		Annotations
			Used for annotation	Used for background	Annotated images	Background images	
05.07.2021	DJI P4P	4.90 to 6.60	9	1	20	2	862
20.05.2022	DJI P4P	4.90 to 5.10	0	10	0	37	0
29.06.2022	DJI P4P	4.80 to 6.00	44	2	131	2	1915
12.07.2022	DJI P4P	5.20 to 5.50	33	0	106	0	2998
13.07.2022	DJI P4P	5.90 to 6.00	33	0	90	0	3029
14.07.2022	DJI P4P	7.20 to 7.60	51	0	97	0	4122
17.08.2022	DJI Matrice 300 RTK*	6.04 to 12.00	30	0	140	0	3325
24.08.2023	DJI P4P	9.90 to 10.00	0	4	0	18	0
30.08.2023	DJI Matrice 300 RTK*	11.98 to 12.02	0	11	0	84	0
05.09.2023	DJI P4P	7.70 to 8.00	0	3	0	19	0
Sum			200	31	584	162	16,251

**Fig. 2:**
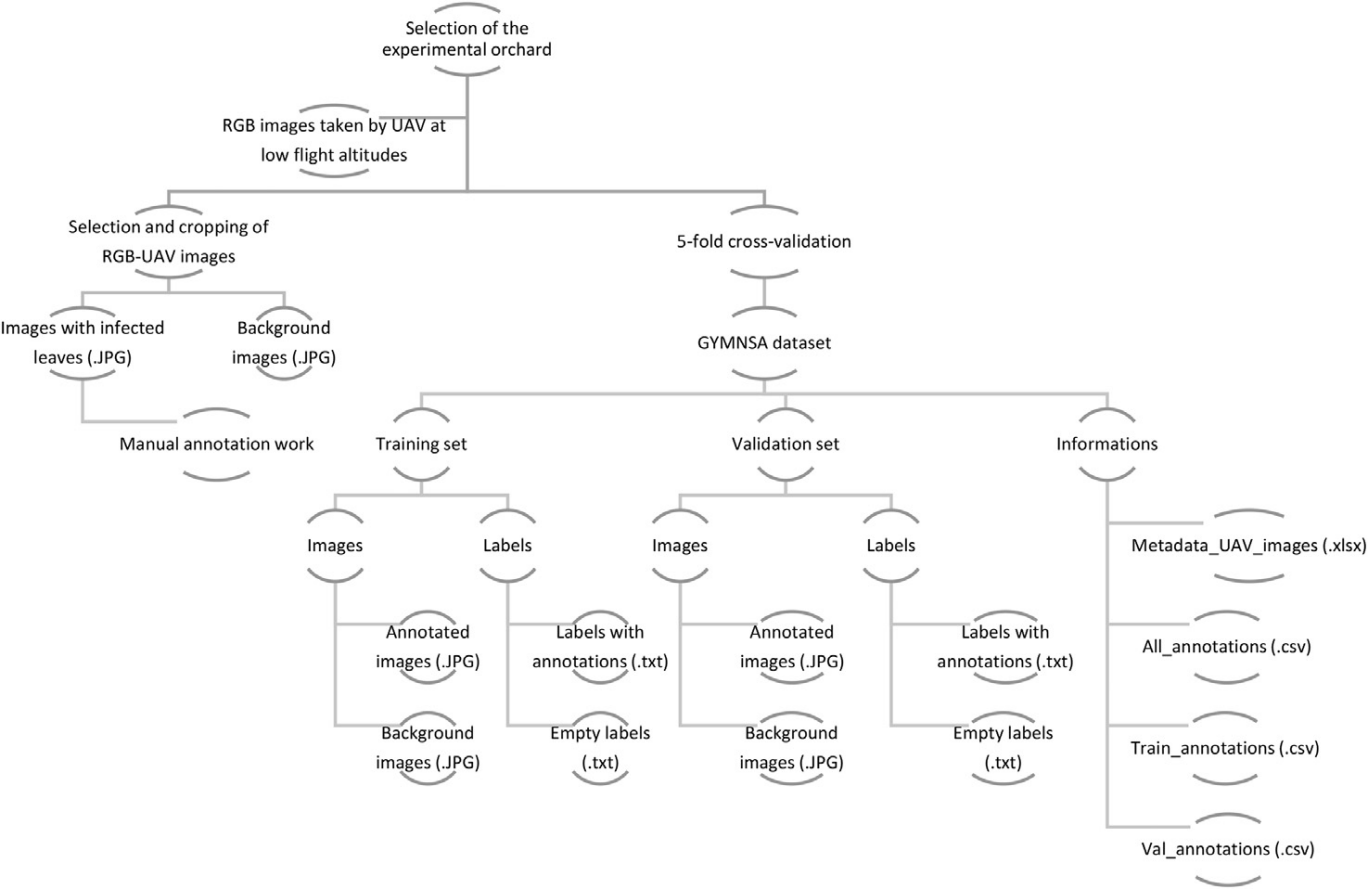
Data collection and folder structure of the GYMNSA dataset.

Each image file of the presented dataset has its corresponding text file in YOLO 1.1 file format. The single-stage model You Only Look Once (YOLO) was chosen because it recognises different objects very quickly and precisely [11]. The following information can be found in the text files of the annotated images in the following order: class_ID, x_centre, y_centre, width, height. There is an empty .txt file for each background image in the respective label folders of the training and validation data. The two folders each contain two subfolders with all the images and labelling (Fig. 2) for training and validating a YOLO model.

The training dataset contains 465 labelled images with 12,591 annotations and 132 background images. The validation dataset contains 119 labelled images with 3660 annotations and 30 background images. In addition to the training and validation folder, an information folder of the dataset is also included. The information folder contains three .csv files in TensorFlow Object Detection format, converted by a Python script [12]. To reproduce the TensorFlow Object Detection format, the YOLO 1.1 file formats were converted to the VOC .xml file format by a Python script [13]. The files Train_annotations.csv (training annotations), Val_annotations.csv (validation annotations) and All_annotations.csv (total annotations) contain the values class, height, width, xmin, ymin, xmax, ymax, which are listed for each file name. Based on the .csv files and the dataset splitting, tf.record files can be created by users to test TensorFlow object detection models in addition to YOLO object detection models. The information folder also provides metadata (Metadata_UAV_images.xlsx) that was extracted using the ExifTool software [2]. This data, such as the flight altitude and gimbal position of the original images, can be used to enable selection or re-sorting of the dataset according to the specific flight parameters.

## 4 Experimental Design, Materials and Methods

### 4.1 Data collection

**Fig. 3:**
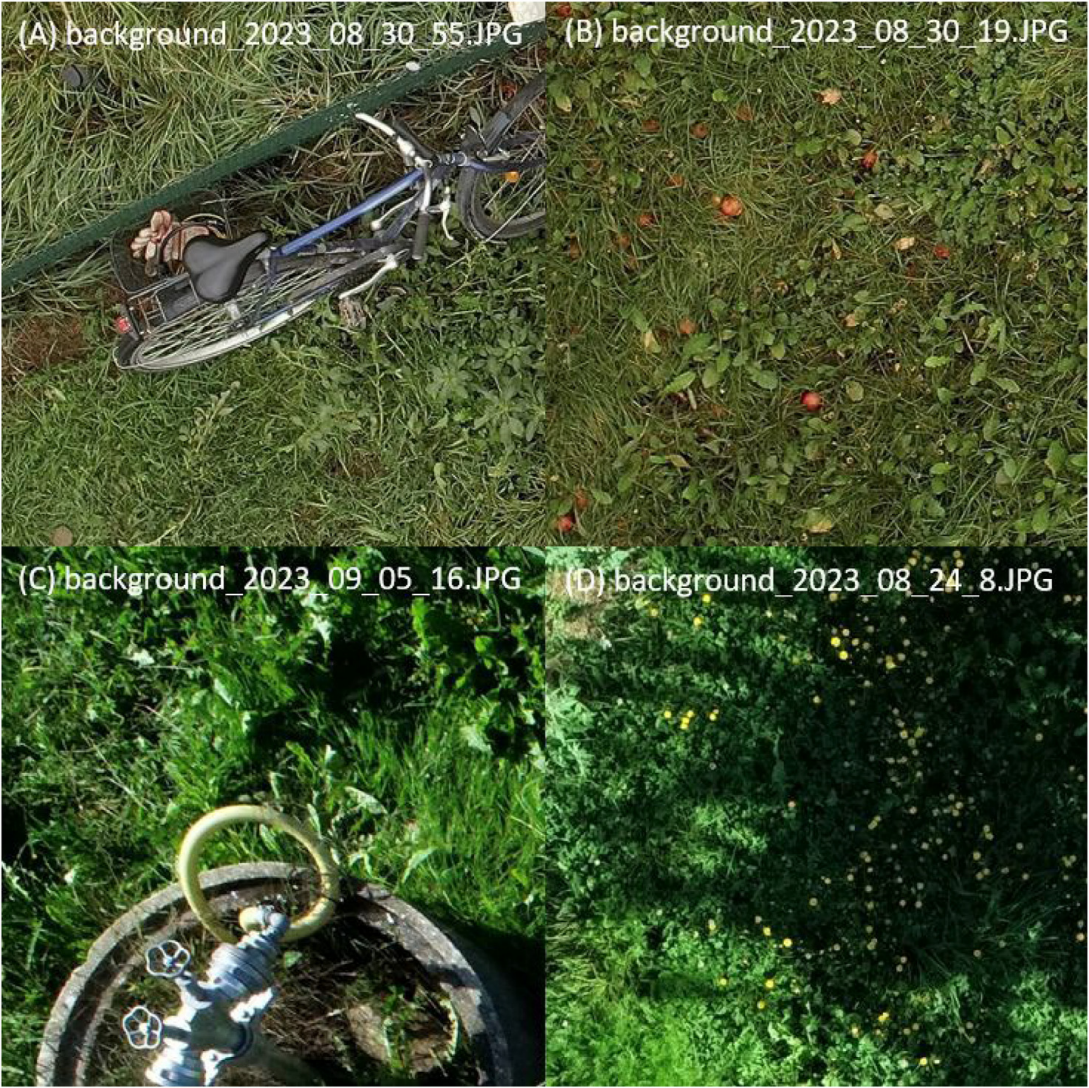
Samples of background images used in the GYMNSA dataset. Taken with the DJI Matrice RTK 300 - Zenmuse P1 © geo-konzept (A, B) and the DJI P4P © Stefanie Reim (C, D).

The UAV RGB raw data was sorted manually after the images were collected. If the images were of good quality and the symptoms of pear rust were clearly recognisable, the images were cropped into smaller RGB images (768  ×  768 pixels) without overlapping using the Pillow Python library [4]. This was followed by a manual pre-selection of image snippets to create datasets for the annotation tool CVAT [1]. Background images were created both from the same flight campaigns and from extra flight campaigns. As a result, a wide variety of possible interference factors was included in this dataset (Fig. 3).

Interfering factors are features in the image that are close in colour and texture to leaves infected with pear rust. For example flowering greenery, fruit or everyday objects on the field can lead to false-positive results and should be reduced using this approach (Fig. 3).

Data collection under different weather and light conditions (Table 2) should also help to ensure robust detection of the trained model. On average, cloud cover was 3 to 8 octa [14,15] and wind speeds between 0.7 and 3.2 m/s were recorded [16]. The light value of the camera systems ranged from +10.3 to +13.6 LV. The ISO varied from 100 to 800 and the shutter speed was adjusted from 1/80 to 1/1000 depending on the flight parameters and weather conditions, such as the light value or exposure value and the f-number.

**Table 2:** GYMNSA dataset overview with weather conditions [[16], [17], [18]], image parameters and average ground sampling distance (GSD). UAV images of pear rust from 2021 to 2023. Image and sensor data from DJI P4P: image width = 5472; image height = 3648; sensor width = 13.2 mm; sensor height = 8.8 mm. Image and sensor data from Zenmuse P1: image width = 8192; image height = 5460; sensor width = 35.9 mm; sensor height = 24 mm.

Date	Drone / Camera-system	Wind speed a [m/s]	Cloud cover c [octa]	Light value [LV]	ISO	F-number	Shutter speed	Ground sampling distance [centimetre/pixel]
05.07.2021	DJI P4P	1.6	6	+11.2 to +12.0	100	4.5	1/120 to 1/200	0.13 to 0.17
20.05.2022	DJI P4P	0.9–1.2	7–8	+12.3 to +13.3	100	5.0–5.6	1/200 to 1/320	0.13
29.06.2022	DJI P4P	2.3–3.2	8	+10.6 to +12.6	100	4.0–5.0	1/100 to 1/160	0.13 to 0.16
12.07.2022	DJI P4P	1.9	3–4	+10.3 to +11.7	100	4.0–4.5	1/80 to 1/160	0.14 to 0.15
13.07.2022	DJI P4P	0.7–1.8	6	+11.7 to +13.6	100	4.5–5.6	1/160 to 1/400	0.14 to 0.16
14.07.2022	DJI P4P	1.2	6	+12.0 to +13.0	100	4.5–5.0	1/200 to 1/320	0.19 to 0.20
17.08.2022	DJI Matrice 300 RTK^b^	1.3–1.7	4–7	+11.9 to +13.6	400 to 800	5.6–7.1	1/1000	0.07 to 0.21
24.08.2023	DJI P4P	0.9–1.1	4–8	+12.3 to +12.6	800	7.1–8.0	1/793	0.27
30.08.2023	DJI Matrice 300 RTK^b^	1.2–1.4	8	+10.3 to +11.0	800	4.0–5.0	1/640	0.21
05.09.2023	DJI P4P	3.0	3	+12.3 to +13.3	400	5.6–8.0	1/636	0.21

a Hourly mean wind speed during the recording period.

b Including high-resolution camera Zenmuse P1.

c The cloud cover was recorded by the Dresden-Klotzsche weather station, which is located about 9 km north-west air distance of the experimental orchard in Dresden-Pillnitz.

The ground sampling distance (GSD) describes the distance between two consecutive pixel centres, measured on the ground. The bigger the GSD value of the image, the lower the spatial resolution of the image and the less detail is visible [17]. The GSD was calculated as follows:

(1) $$GSD\;\left[cm/px\right]=\frac{\left(Sensor\;width\;\left[mm\right]\;*\;Relative\;altitude\left[m\right]\;*\;100\right)}{\left(Focal\;lenght\;\left[mm\right]\;*\;Image\;width\;\left[px\right]\right)}$$

### 4.2 Annotation work and dataset

**Fig. 4:**
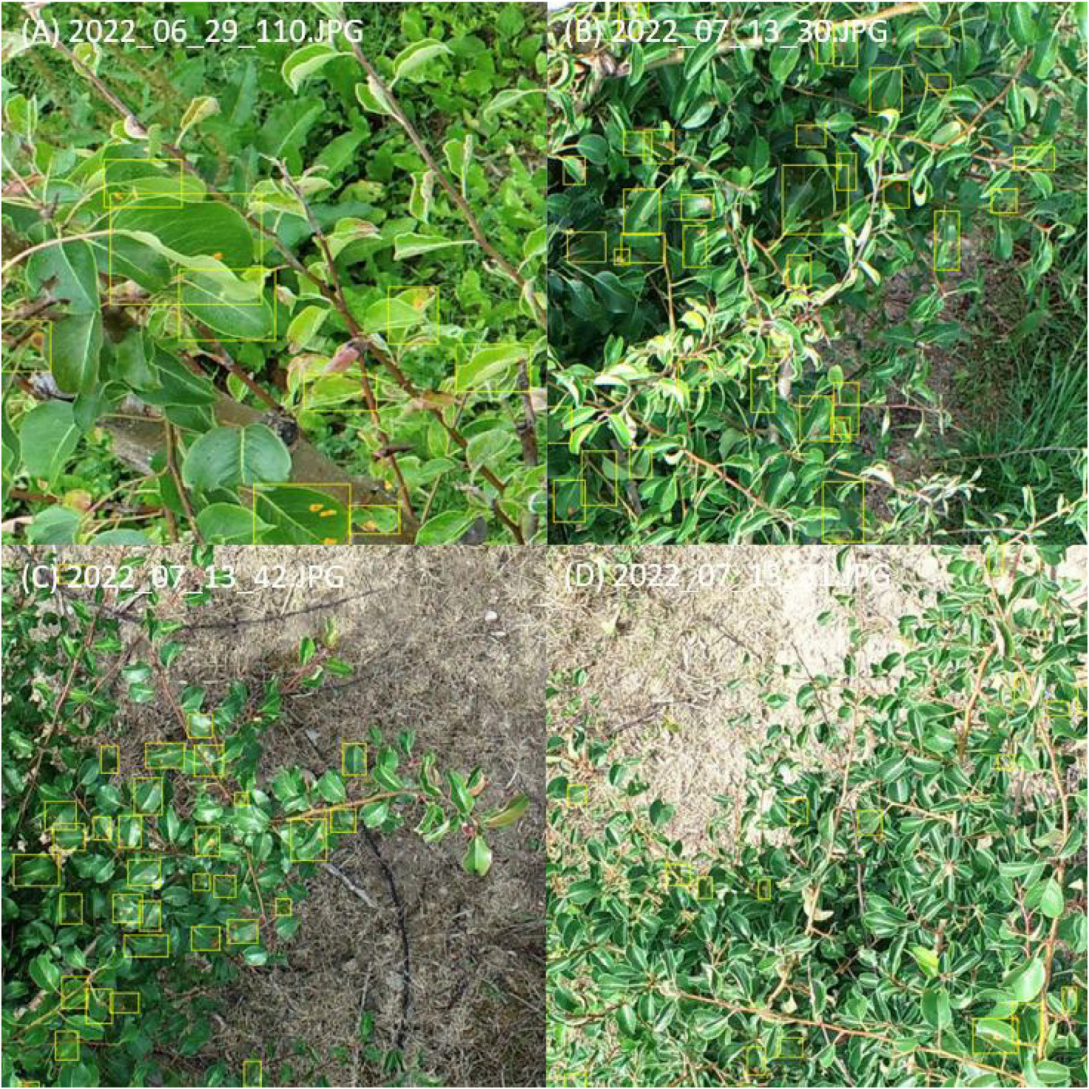
Samples of annotated images with pear rust infected leaves used for the dataset. Taken with the DJI P4P © Stefanie Reim (A-D).

The symptom labelling of the cropped UAV RGB images was done manually with the annotation tool CVAT [1]. The 2-point bounding box annotation was selected as the labelling method. Rectangular bounding boxes were drawn around the leaf area with pear rust symptoms. For each bounding box of the class ‘GYMNSA’, i.e. a leaf infected with *Gymnosporangium sabinae*, the class_ID (0 = GYMNSA) and the coordinates of the bounding box in the image (x_centre, y_centre, width, height) are saved in the associated label file, here in YOLO 1.1 file format. The labelling of pear leaves infected with pear rust involved five different stages of infection (Fig. 1). All infection stages were labelled as ‘GYMNSA’ (= infected). Both whole infected leaves and overlapping infected leaves were labelled. Infected leaves of pear rust were also annotated if they showed strong light reflections in addition to the infection (Fig. 4). After the annotation work was completed in CVAT, a labelling file (.txt) in YOLO 1.1 file format was generated for each image, which can be used for training object detection models such as YOLO.

Using 5-fold cross-validation with the configuration shuffle = True and random_state = 42, the best split of the dataset was selected and the annotated images and background images were divided into two folders for training and validation (Table 3). The configuration shuffle = True, in relation to the GYMNSA dataset, caused the image datasets from the different flight campaigns or acquisition days to be mixed. This meant that every stage of pear rust infection and different flight parameters could be included in the training and validation dataset. The configuration random_state = 42 influences the order of the indices, controls the randomness of the individual folds and serves the reproducibility of the dataset mixture and the splitting of the GYMNSA dataset [18].

**Table 3:** Training and validation splits as well as annotated images and background images in relation to the total dataset.

Dataset split	Annotated images	Background images	Sum images	Ratio images	
				Annotated	Background
Training	465	132	597	77.89%	22.11%
Validation	119	30	149	79.87%	20.13%
Sum images	584	162	746	78.28%	21.72%
Ratio split	Training	79.62%	81.48%	80.03%	
	Validation	20.38%	18.52%	19.97%	

## Limitations

Not applicable.

## Ethics Statement

For this research and analysis, no human or animal subjects were used and no data from social media platforms was used. The authors confirm that the provided dataset and presented work strictly meet the ethics requirements for publication in Data in Brief as mentioned in https://www.elsevier.com/de-de/researcher/author/policies-and-guidelines.

## CRediT Author Statement

**Virginia Maß:** Software, Methodology, Investigation, Validation, Data Curation, Visualization, Writing - Original Draft; **Stefanie Reim:** Project administration, Conceptualization, Funding acquisition Supervision, Resources, Investigation, Validation, Visualization, Writing - Review & Editing; **Pendar Alirezazadeh:** Software, Methodology; **Johannes Seidl-Schulz:** Software, Validation, Methodology, Supervision, Visualization, Investigation; **Matthias Leipnitz:** Conceptualization, Methodology; **Eric Fritzsche:** Resources, Investigation; **Rasheed Ali Adam Ibraheem:** Software, Investigation; **Martin Geyer:** Supervision, Funding acquisition; **Michael Pflanz:** Conceptualization, Funding acquisition.

## Data Availability

Mendeley DataGYMNSA dataset (Original data).
